# Uncertainties in Model-Based Diesel Particulate Filter Diagnostics Using a Soot Sensor

**DOI:** 10.3390/s19143141

**Published:** 2019-07-17

**Authors:** Dimitrios Kontses, Savas Geivanidis, Pavlos Fragkiadoulakis, Zissis Samaras

**Affiliations:** Laboratory of Applied Thermodynamics, Aristotle University of Thessaloniki, Administration Building, University Campus, P.O. Box 458, GR-54124 Thessaloniki, Greece

**Keywords:** resistive soot sensor, DPF failure, OBD, soot model, DPF model, sensor model, OBD algorithms, error propagation

## Abstract

Monitoring the filtration efficiency of the diesel particulate filter (DPF), is a legislative requirement for minimizing particulate matter (PM) emissions from diesel engines of passenger cars and heavy-duty vehicles. To reach this target, on-board diagnostics (OBD) in real-time operation are required. Such systems in passenger cars are often utilizing a soot sensor, models for PM emissions simulation and algorithms for diagnosis. Their performance is associated with a series of challenges related to the accuracy and effectiveness of involved models, algorithms and hardware. This paper analyzes the main influencing factors and their impact on the effectiveness of the OBD system. The followed method comprised an error propagation analysis to quantify the error of detection during a New European Driving Cycle (NEDC). The results of the study regarding the performance of the OBD model showed that the total error of diagnosis is ±28%. This performance can be improved by increasing the sensor accuracy and the soot model, which can make the model appropriate for even tighter legislation limits and other approaches such as on-board monitoring (OBM).

## 1. Introduction

Air pollution is recognized as a serious and worldwide concern for human beings and the natural environment [1,2]. Premature mortality due to several deceases is related to low air quality in rural and urban environments on a global scale [3]. Particulate matter (PM) emissions, notably, are considered one of the most harmful pollutants [4,5]. Transportation sector and road vehicles are historically one of the primary sources of pollutants, including PM emissions [6].

One of the most significant current discussions for countries and authorities is the measures that need to be adopted to reduce the PM emissions from internal combustion engines of road vehicles [7]. The current stringent legislation in the European Union (EU) and the United States (US) for PM emissions from a diesel engine has led to installation of a diesel particulate filter (DPF) in the exhaust aftertreatment (EAT) system of modern diesel vehicles [8,9]. In addition, the implementation of a real-time monitoring algorithm of the filtration efficiency of the DPF on the on-board diagnostics (OBD) system is necessary. This study focuses on the EU legislation and requirements to detect any malfunction of the system before the PM emissions exceed the mass limit of 12 mg/km during a specific legislative driving cycle and the malfunction indication light (MIL) illuminates [9,10]. Also, the total removal of the DPF must be diagnosed. The EU OBD requirements are summarized and compared to the EU emission limits in Table 1.

**Figure 1:**
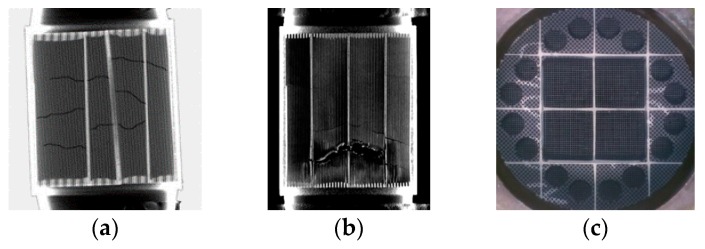
(**a**) Cracks on DPF substrate; (**b**) melted DPF; (**c**) partial unplugged DPF.

In real applications, this malfunction, which results in low filtration efficiency and high PM emissions can be attributed to the following three circumstances [11] (Figure 1):

- Cracks in the substrate of the DPF: Ring-off cracks or internal cracks can occur due to thermal stresses on the substrate by uncontrolled regenerations or by drop to idle during DPF regeneration [12].
- Melting of the substrate: Although the melting point is safely high for silicon carbide (SiC) substrates (2800 °C), cordierite substrates are prone to melting in uncontrolled regenerations due to a lower melting point (1200 °C) [13].
- Unplugged DPF or total removal: A damaged DPF, the high pressure-drop due to ash accumulation, the fuel penalty of active regeneration and engine tuning reasons, can lead vehicle owners to remove or destroy the DPF (DPF tampering) instead of replacing it [14].

All these cases result in a reduced filtration efficiency, and depending on the legislation in question, the risk of exceedance or violation of the legislative limits.

A damaged or removed DPF substrate can be identified by the pressure drop method, which relies on the measurement of the difference in the exhaust gas pressure upstream and downstream of the DPF. Using the appropriate software and algorithms, a diagnosis is possible. This method, however, is reaching its limits in terms of accuracy and robustness of the diagnosis [15], as the legislation limits are becoming lower.

PM sensors fitted into the exhaust line aided by OBD models have recently been used for the diagnosis of a failure in the DPF. Various sensor technologies have been tested or are currently developed by several manufacturers as described and compared in recent publications [16,17,18,19,20]:

- Radio-frequency (RF): The accumulated soot in a DPF absorbs and changes the microwave signal produced by an RF antenna. A correlation of the signal change with the soot accumulation is possible [21].
- Electrical charge-based sensors: Diffusion charger (DC) technology, which relies on measuring the corona induced charge of particles [22,23] and electrostatic sensors which measure the amplified natural charge of the particles [24], are considered the most suitable electrical charge-based sensors for DPF diagnosis.
- Optical sensors: Optical sensors are based on the result of the interaction between the exhaust particles with a laser beam. Despite the domination of optical devices for laboratory and (Portable Emissions Measurement System (PEMS) applications, the maturity level of sensors for OBD applications is considered low. Laser induced incandescence (LII) sensors [25,26] are promising solutions for future advanced sensors.
- Resistive sensors: The high conductivity of soot particles accumulated in dendritic geometry between two electrodes reduces the resistance of the system [27]. Below a specific value of the resistance the accumulation mode is over, and the regeneration mode of the sensor is activated to clean the soot deposits, increasing the value of resistance to infinite. The critical measurable quantity of a soot sensor is the duration of the sensor’s accumulation event, which is called the response time. This signal can be correlated with the soot emissions based on a statistical model or a physical model [28].

Six years ago, experiments on pre-production “spark-plug sized” soot sensors [29] revealed the progress made in this area and depicted the different levels of development and accuracy for each sensor and technology.

Focusing on the resistive soot sensors, along with the appropriate sub-models and algorithms, they can offer enhanced performance in DPF diagnosis and are currently the common solution for DPF failure detection [15,18]. Several studies have presented the development of prototypes based on resistive technology [30,31,32,33,34,35]. Nowadays, despite the high maturity level, the low cost and the broad adoption of resistive sensors in passenger car applications, the DPF diagnosis based on this technology and the necessary sub-models, is still a complex solution with a lot of challenges [36]. This inevitably leads to limited accuracy and capability of identifying different DPF failure levels which might not be a significant issue for the already used OBD applications but makes it prohibitive to use the sensor and the model for future more stringent regulations or other approaches such as on-board monitoring (OBM). In that respect, and also considering the low availability of publicized results for the performance of the overall OBD model, this paper presents and describes in details the involved models and algorithms, compares the different approaches in every step and calculates the propagated errors starting from the sensor signal and getting through the final DPF diagnosis based on an example of a New European Driving Cycle (NEDC). The findings should make an important contribution to the understanding of different approaches and the quantification of the error and the inaccuracy of all steps during a model-based DPF diagnosis.

## 2. Materials and Methods

### 2.1. Engine and Exhaust Aftertreatment

**Figure 2:**
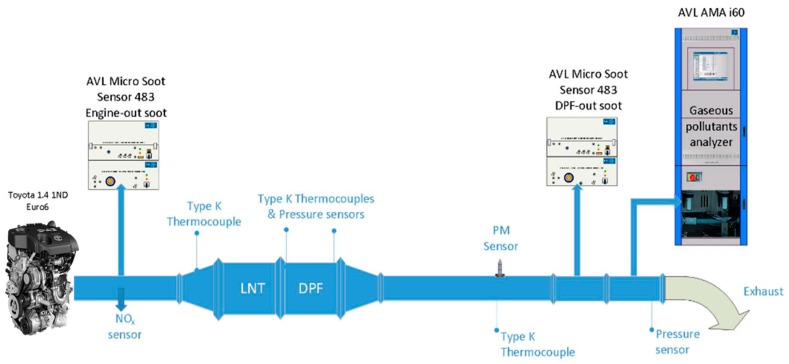
Test engine set-up with exhaust gas analyzers and sensors.

The evaluation of the OBD models and the calculation of errors were performed using data from a Euro 6-compliant Toyota 1.4 L diesel engine (Table 2) with a modified exhaust line (Figure 2) coupled with a transient dynamometer in a laboratory test bench. The original NO_x_ exhaust aftertreatment (lean NO_x_ trap, LNT) was used. The DPF was replaced with artificially unplugged SiC uncoated DPFs. Based on the plug-removal technique for reducing the DPF filtration efficiency and additional try and error experiments, 3 DPFs were created for the needs of this study:

- “OTL DPF”: PM emissions on cold NEDC at the OBD threshold limit (OTL) = 12 mg/km
- “Threshold DPF”: PM emissions on cold NEDC = 9.7 mg/km
- “TA DPF”: PM emissions on cold NEDC at the type approval (TA) limit = 4.5 mg/km

In total, 1750 plugs were symmetrically removed from the outlet of the DPF for the OTL DPF, 1420 plugs for the threshold DPF and 950 plugs for the TA DPF.

PM emissions were determined by correlation to soot concentration measured on a second-by-second basis using an AVL 483 Micro Soot Sensor (MSS). The accuracy of the correlation of soot to PM was high enough to allow the good performance of the final model and implementation since the same inaccuracy is introduced to all emission levels.

Two MSSs were used to measure engine-out and DPF-out soot concentration. AVL AMA i60 was used to measure gaseous pollutant concentrations for validating the correct and repeatable operation of the engine compared to a reference measurement. A resistive soot sensor was installed downstream of the DPF at an adequate distance to ensure fully developed exhaust flow. Exhaust temperature was measured at the sensor location to be used as input on the physical model. Exhaust velocity was calculated from the intake air flow measured by the mass air flow (MAF) sensor of the engine in combination with the fuel injection quantity calculated by the engine control unit (ECU). This allows the implementation of the model to an ECU using existing sensors and signals.

### 2.2. Driving Cycles

**Figure 3:**
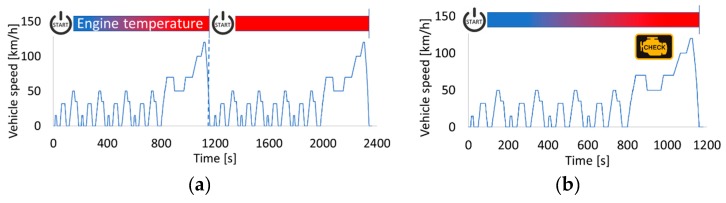
EU OBD legislation (**a**) first and second preconditioning cycles; (**b**) emission tests cycle (example of NEDC).

**Figure 4:**
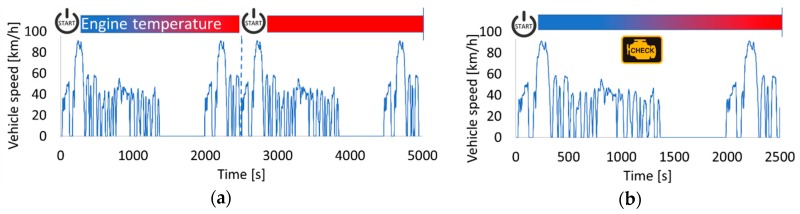
US OBD legislation (**a**) first and second preconditioning cycles; (**b**) emission tests cycle (example of Federal Test Procedures (FTP-75)).

In order to be in line with certification procedures for approval of compliance of a vehicle to OBD requirements in both EU and US legislation, the test protocols comprise two parts (for the current study, the NEDC was selected to calculate the errors and demonstrate the accuracy of the OBD model):

- The first part consists of a preconditioning period which for the EU is two consecutive NEDCs (or Worldwide harmonized Light-duty Test Cycles (WLTCs)) and for the US two Federal Test Procedures (FTP-75), Supplemental Emissions Tests (SETs) or Unified Cycles (an additional preconditioning cycle is possible upon request).
- The second part is the exhaust emission test. This is a cold cycle, and at least six hours of soaking are needed before its start. Prior to the end of the emission tests cycle (or before the engine stop for the FTP-75 cycle), a vehicle set with the criteria limits for the DPF diagnosis must diagnose the malfunction and illuminate the MIL (Figure 3 and Figure 4).

### 2.3. Resistive Soot Sensor

**Figure 5:**
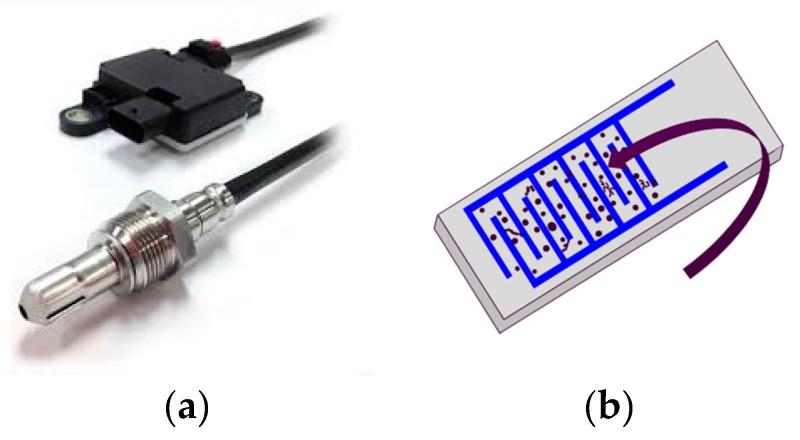
(**a**) Resistive soot sensor (Stoneridge Inc.) with its electronic control module (ECM) and sensor probe; (**b**) sensor element with soot particles (enlarged).

The current analysis was based on test results from commercially available resistive soot sensors provided by Stoneridge Inc. (Novi, MI, USA). These sensors consist of a sensor probe and the electronic control module (ECM) (Figure 5) necessary for control and communication with the ECU of a vehicle. The probe of a sensor comprises the sensor tip to condition the flow before it reaches the sensing element contained inside the sensor probe. This element is a ceramic plate from aluminum oxide and is installed vertically to the exhaust tube. Platinum electrodes are fitted on the ceramic plate at a specific distance between each other and create the sensing area.

### 2.4. OBD Model and Calibration of the Threshold Limit

**Figure 6:**
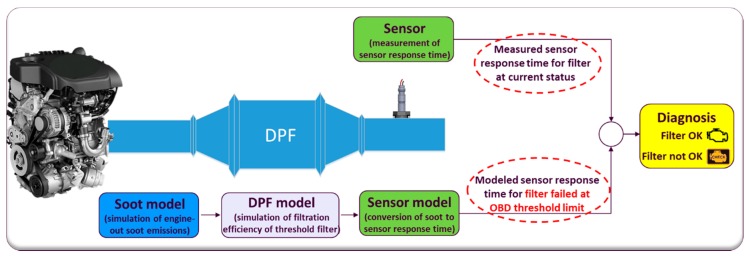
Overview of the OBD model developed in Matlab Simulink©.

The core of this analysis is the OBD Model that was developed using the commercial software MATLAB Simulink© (version 8.4, R2014b). This model, as illustrated in Figure 6, consists of the signal of the soot sensor (sensor box), the OBD algorithms which execute the final DPF diagnosis and three sub-models described below.

The concept is to check if due to a DPF failure, the actual PM emissions of the vehicle are higher than the OTL. The legislation requires this check to be performed for the evaluation of the effectiveness of the DPF over a legislative driving cycle (NEDC in this study). Emissions higher than 12 mg/km over NEDC should turn the MIL on.

This is achieved by comparing the actual sensor response time reported by a sensor during an NEDC (1*st* branch in Figure 6) with the simulated response time when the PM emissions are at the OTL (2*nd* branch):

- Higher measured response time means lower than the OTL emissions (DPF OK)
- Lower measured response time indicates higher than the OTL emissions (DPF not OK)

#### 2.4.1. Soot Model

This model simulates the engine-out soot emissions produced as a function of variables available in the ECU. For OBD applications, a map-based model using the engine speed and torque (or injected fuel mass) as inputs is the default approach offering adequate accuracy with low computational demands. For improved performance and accuracy, the steady-state base map was corrected for transient operation based on additional measurements according to the methodology presented by Neumann et al. [37]. For this study, the transient correction was based on three influencing parameters: intake air flow, rail pressure and injection timing. The main equation for the corrected soot concentration is:

(1) $$C_{Soot}\left(N,m\right)=C_{0}\left(N,m\right)+\overset{n}{\underset{k=1}{\sum }}\frac{\partial C_{0,k}}{\partial Z_{k}}\left(N,m\right)\times \left[Z_{k}\left(N,m\right)-Z_{0,k}\left(N,m\right)\right],$$

where,

- N: engine speed;
- m: injected fuel mass;
- $C_{Soot}\left(N,m\right)$: corrected soot concentration at a specific operating point during transient operation;
- $C_{0}\left(N,m\right)$: soot concentration from base emission map (steady-state);
- $\sum_{k=1}^{n}\frac{\partial C_{0,k}}{\partial Z_{k}}\left(N,m\right)\times \left[Z_{k}\left(N,m\right)-Z_{0,k}\left(N,m\right)\right]$: soot concentration from transient correction;
- *k*: influencing parameters for transient correction;
- $\frac{\partial C_{0,k}}{\partial Z_{k}}$: partial derivative of soot concentration against each influencing parameter;
- $Z_{k}\left(N,m\right)$: value of the influencing parameter at a specific operating point during transient operation;
- $Z_{0,k}\left(N,m\right)$: value of the influencing parameter at a specific operating point during steady-state operation.

**Figure 7:**
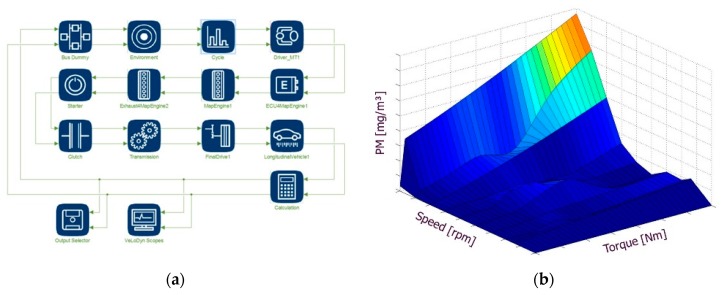
(**a**) Overview of the VeLoDyn model for simulation of engine-out soot emissions; (**b**) soot map used for calculation of engine-out soot emissions in every time step according to engine speed and torque which are provided by the vehicle’s engine control unit (ECU).

Additionally, by using IAV/Exothermia VeLoDyn software (version 2017) as a part of the Matlab© library, it is possible to simulate the emissions along with the transient corrections of a vehicle under different legislative test cycle or real-world driving profiles. VeLoDyn calculates soot emissions for a given driving profile using a combination of static soot emission maps, vehicle and engine operation variables, environmental conditions, road load, etc (Figure 7). Therefore, VeLoDyn performs sensitivity analyses on different vehicles or driving patterns.

#### 2.4.2. DPF Model

**Figure 8:**
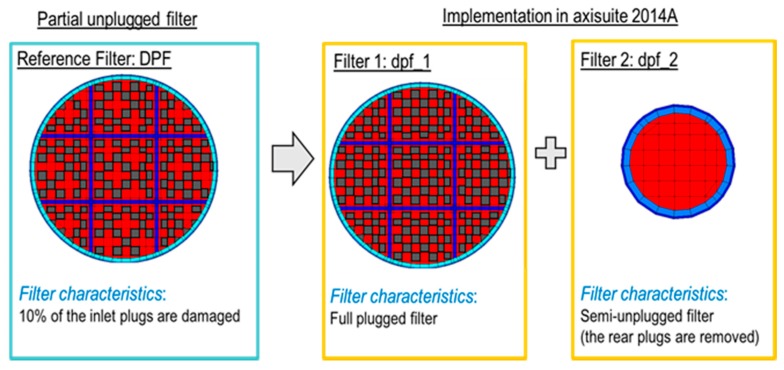
Simulation of a partial unplugged DPF (Axisuite).

The DPF model calculates the filtration efficiency of a DPF failed at the threshold limit and provides the DPF-out soot emissions to be used as input to the sensor model. The standard practice for simple DPF models is to use a constant filtration efficiency calculated based on measurement of soot concentration upstream and downstream of a compromised DPF during the NEDC. An example of an alternative advanced DPF filtration efficiency model is the Exothermia/Axisuite (version 2017A) partial unplugged DPF model, which was also used in the current study. The model consists of two DPFs connected in parallel in the exhaust. The first DPF (filter 1) is a full plugged DPF (filtration efficiency almost 100%) and the second DPF (filter 2) is a fully unplugged DPF (filtration efficiency 0%) (Figure 8). In terms of simulation, the two filters are identical except for the length of outlet plugs of filter 2 which is set to zero, and the diameters which are calculated based on the number of removed plugs and the number of cells per square inch of the filter (cpsi). This DPF model can accurately and robustly determine the filtration efficiency in a second-by-second basis.

The accurate simulation of Axisuite models demands some calibration effort. The procedure consists of three calibration targets:

- Soot and substrate parameters
- Soot compressibility model parameters
- Filtration parameters

This calibration procedure is the same for all Axisuite DPF models and is described in detail in a previous publication [38]. All calibration steps were performed in the same way for the current study.

Although the Axisuite model is hard to be implemented in the current ECUs, it is a valuable tool for offline calculations to demonstrate the feasible accuracy of a DPF model. Also, a compromise between these two solutions could be models which are based on the exhaust flow or temperature for the calculation of the second-by-second efficiency [36] but are not included in the current analysis.

#### 2.4.3. Sensor Model

**Figure 9:**
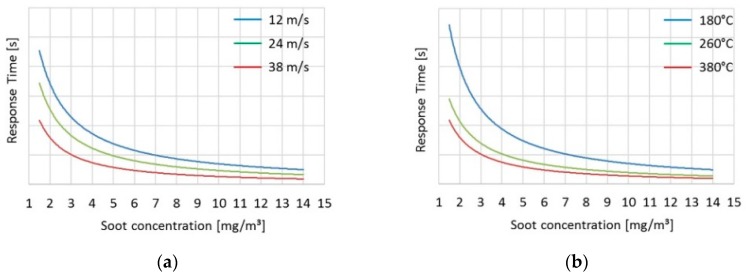
Example of performance of the statistical model over different levels of soot, velocity and temperature. (**a**) Sensor response time at 260 °C for three exhaust gas velocity levels (**b**) Example of sensor response time at exhaust speed of 24 m/s for three exhaust gas temperature levels.

The measured value of the sensor, as discussed previously, is the response time, and thus, the sensor model should also yield the same quantity. This is usually done with a statistical model. A physical model, as presented in a previous publication [28], can be used as an alternative approach to the statistical model but is not investigated in the current study. For the statistical model, a mathematical expression or empirical function is typically generated with statistical regression, using measured sensor responses over steady-state points defined by a design of experiment (DOE) covering a wide range of engine operations. For the needs of the current study, a function with three inputs, namely exhaust soot concentration, exhaust gas velocity and temperature were used. The performance of a typical statistical model is depicted in Figure 9 for three different levels of exhaust temperature and velocity at different soot values.

#### 2.4.4. OBD Algorithms

The direct comparison of the measured and modeled response times at the end of a sensor accumulation period is the simplest method as described above but requires full sensor accumulation periods which can take several minutes for a slightly damaged DPF. On the other hand, an indirect comparison at every time step can increase the frequency of detection. In this case, the OBD index is calculated at every time step (usually dt = 1 s) as in [36]:

(2) $$OBDIndex\left(t\right)=\overset{t}{\underset{0}{\int }}\frac{1}{\tau \left(t\right)}dt,$$

where,

- t = duration from the start of accumulation (input from the sensor, 1st branch in Figure 6);
- τ(t) = estimated response time (input from the models, 2nd branch in Figure 6).

For an OTL DPF (12 mg/km over NEDC) an “ideal” OBD model and an accurate sensor yield an OBD index = 1 at the end of sensor accumulation and the DPF should be diagnosed as not OK. In summary, for all emission levels during an NEDC:

- ≥12 mg/km → OBD index ≤ 1 → DPF not OK
- <12 mg/km → OBD index > 1 → DPF OK

**Figure 10:**
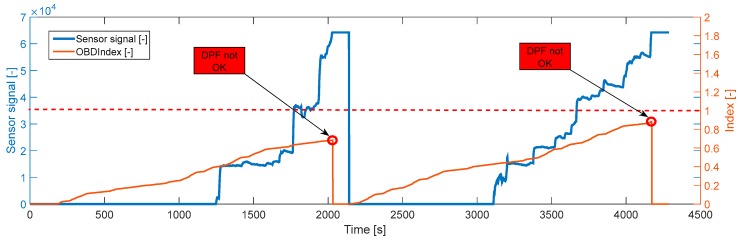
OBD index and sensor signal across a transient operation for an above OBD threshold limit (OTL) DPF. Both sensor accumulation periods result in a correct diagnosis of the failed DPF.

Figure 10 illustrates an example of successful “DPF not OK” diagnosis during transient operation with an artificially destroyed DPF above the OTL (13.5 mg/km).

During real-world operation, it is possible for a failed DPF at emission levels above the OTL (e.g., exceedance of 1 mg/km), to lead to OBD index >1 and thus cannot be correctly diagnosed. This may appear during real-world operation due to the sensor or model being faced with rare, but possible, operating conditions that were not predicted during model setup. This situation though is not acceptable since, according to the legislation, no false pass (error of omission, type II error) is allowed. Therefore, detection should be designed to be performed against a threshold lower than the OTL of the legislation, to serve as a safety margin, ensuring no error of omission under any operating conditions. This level is set between the OTL (12 mg/km) and the TA limit = 4.5 mg/km. In addition, OBD system design targets the minimization of the number of false failures (error of commission, type I error) according to the industrial standards (close to zero errors of commission) because otherwise, it would lead to unnecessary costs for technical inspection and customers complaints. In this case, the threshold should be as close to the OTL (12 mg/km) as possible. Therefore, a compromise between the two design competing targets is necessary for real-world applications and the model and the efficiency need to be calibrated with respect to this requirement. The final threshold emissions should be as close to the OTL as possible according to the above theory (e.g., 9 mg/km). To adjust this limit, usually, the threshold filtration efficiency of the DPF is calibrated.

**Figure 11:**
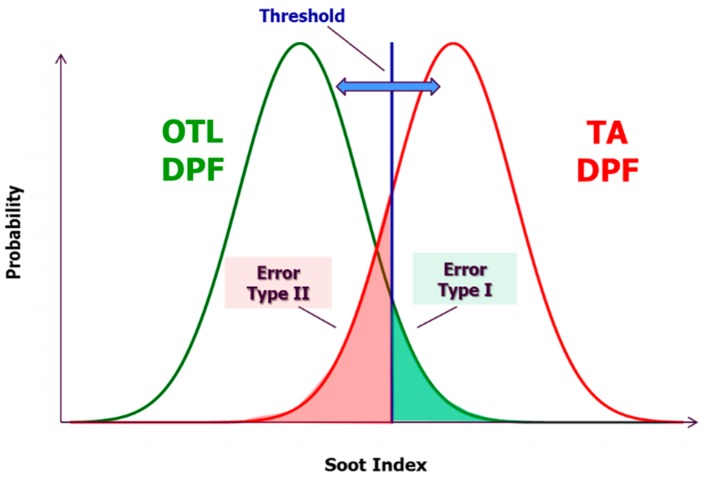
Definition of threshold OBD index, and type I and II errors as calculated from the measurements of an OTL and a type approval (TA) DPF. The target is to find a compromise between low Type I and Type II errors.

For setting up the model presented in this paper, one should execute the following calibration steps:

- Measure the sensor response times for the OTL and TA DPFs on different legislative driving cycles on the test bench. For the final calibration of a series production vehicle, additional on-road measurements must also be used.
- Calculate the OBD Indexes for all sensor accumulations using the filtration efficiency for 12 mg/km over NEDC initially.
- Calculate the threshold index, which allows minimum errors of type I and II (Figure 11). According to the needs of each calibration campaign, the threshold can be moved towards the direction of zeroing either the type I or type II error. In most cases, the priority is to ensure zero type II errors (legislation need), and thus, the index threshold is moved towards lower values.
- Calibrate the threshold filtration efficiency, which brings the threshold OBD index to 1. This step is not necessary for the case of the constant filtration efficiency but can improve the accuracy of the filtration profile from the Axisuite or similar second-by-second models. Based on this threshold filtration efficiency, the threshold emissions on NEDC can also be calculated as a final reference value.

### 2.5. Error Propagation Analysis

The implementation of the soot, DPF and sensor models in the OBD model, introduces errors closely associated with the input parameters and the method of execution of every sub-model. To separate, quantify and evaluate these errors, an error propagation analysis was performed. The individual errors, as described in the following tables, were calculated based on Equation (3).

In general, if *q* = *f*(*x*, *y*,…), *δx* is the error associated with variable *x*, *δy* the error related to variable *y* and *δq* the requested error, then:

(3) $$\delta q=\sqrt{{\left(\frac{\partial f}{\partial x}*\delta x\right)}^{2}+{\left(\frac{\partial f}{\partial y}*\delta y\right)}^{2}},$$

where $\frac{\partial f}{\partial x}$ is the partial derivative of function *f*(*x*, *y*,…) with respect to the variable x and cyclical with respect to the other variables.

This part of the work evaluates the error propagation through the OBD model in a single sensor accumulation period over a type-approval NEDC.

## 3. Results and Discussion

### 3.1. Error Associated with the Soot Model

In this section, the capabilities of an advanced soot model that involves transient correction over static emissions maps, is compared against the simple option of static soot emission maps only. The method is described in detail in a previous publication [37].

**Figure 12:**
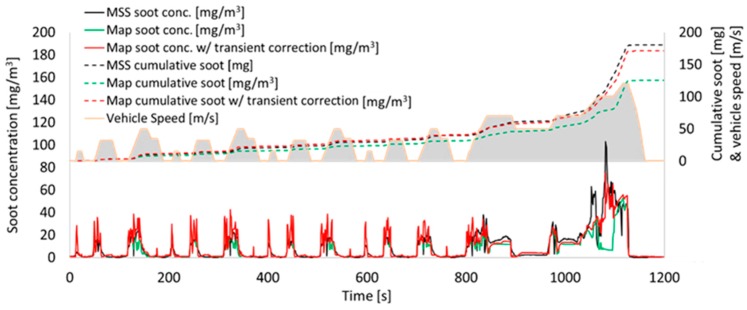
Example of an NEDC. Soot models (base map and base map with transient correction) compared to micro soot sensor (MSS) measurement. The base model is improved with the transient correction.

This model was based on a 3D soot-emission map (base map) enhanced by an additional transient correction. The inputs from the engine ECU or a model (e.g., VeLoDyn) are engine speed and injected fuel mass. The model estimates the soot emissions at every time step of the NEDC, which was used for all demonstrations in the current study. The measurements were performed on the Toyota engine using the engine-out MSS as a reference device. Based on the design of experiment technique, a domain of 56 points was created and measured on the test engine (Table 3). The resulting base soot map was evaluated against the reference measurement device, and the associated error was calculated to be ±25% in reference to cumulative soot (Figure 12) (Table 4). This discrepancy is relatively low compared to the results of a previous study, which estimated three times higher errors [37]. This can be possibly attributed to the better calibration of the test engine. Also, the moderate linear acceleration profile of the NEDC facilitates the good performance of the model. The inaccuracy is expected to be higher at real-world driving patterns. A transient correction was implemented using additional measurements for the calculation of individual partial derivatives of three influencing parameters: intake air flow, rail pressure and injection timing. The final emission profile of the model (base map and transient correction), was almost identical with the MSS measurement with 2% deviation in the cumulative soot value (Figure 12), (Table 4). For this application, no further improvement and calibration through polynomial fit were necessary, as was proposed by Neumann [37].

The main cause of this inaccuracy should be attributed to transient operation. Under these circumstances, there are significant turbocharger lag, mass and air transfer delays, and in a parallel thermal transient phase. The advanced model gives better accuracy over the NEDC and is expected to improve the performance of the soot model for all driving conditions.

Nevertheless, the transient correction demands a lot of calibration effort for every application. Another possible future alternative for accuracy improvement is the calibration of the model based on several data from real driving operation of the vehicle [36]. Also, the utilization of additional input parameters could enhance the accuracy without the need for exhaustive calibration. An example of this scheme is the signal of cheap cylinder pressure sensors [39].

In terms of accuracy, the worst-case scenario for the NEDC (without the transient correction) was selected for error propagation calculations.

### 3.2. Error Associated with the DPF Model

The DPF model is calibrated to simulate the filtration efficiency of a failed DPF that would lead to vehicle emissions over NEDC at the OTL, considering the engine-out soot emissions estimated by the soot model. For the current study, three legislative cycles (NEDC, WLTC, FTP-75) were run for the two selected emission levels (TA and OTL) to calibrate and choose the threshold filtration (two repetitions of every cycle, 12 cycles in total). All cycles were run with the same preconditioning procedure (two NEDCs) for the stabilization of DPF efficiency. Following the calibration steps described in the methodology section, the threshold filtration efficiency was set at 60%, which for our engine, corresponds to 9.7 mg/km over a NEDC. This result suggests that a DPF with PM emissions on NEDC above this limit should trigger the MIL.

**Figure 13:**
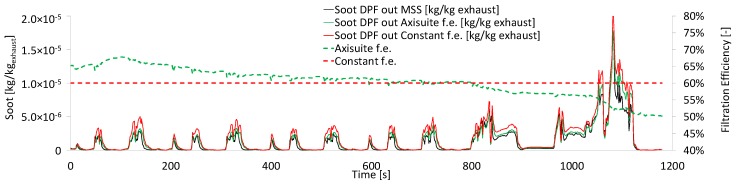
DPF-out soot emissions calculated based on the constant filtration efficiency and the filtration efficiency calculated by the Axisuite model. The comparison to the MSS measurement, reveals the advantage of the Axisuite model.

Advanced models (e.g., Axisuite) can provide a more accurate second-by-second efficiency profile. For the specific engine and DPF, the threshold DPF with 9.7 mg/km emissions was created by symmetrically removing 1420 plugs from the DPF outlet. The DPF filtration efficiency profile for the NEDC is presented in Figure 13. The average value of the Axisuite efficiency profile is similar to the constant filtration efficiency. The DPF-out soot based on Axisuite is closer to the measurement of the MSS. This improvement compared to soot DPF-out based on constant filtration efficiency, is a result of the more accurate second-by-second prediction of the filtration efficiency. The reason for this behavior is that the model accounts for the soot loading of the DPF and the effect of exhaust flow. A few publications are focusing on the filtration efficiency of unplugged DPFs [40,41], but there is no available data for transient operation. Therefore, it is not possible to compare or validate the results of this study.

**Figure 14:**
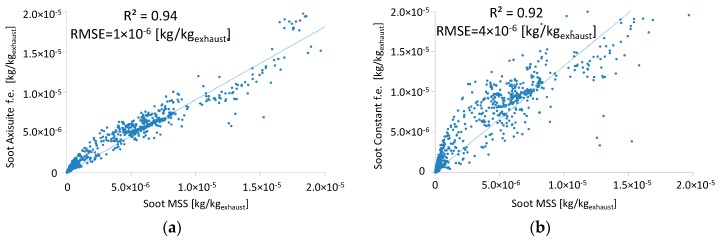
Modeled DPF filtration efficiency (**a**) calculated by Axisuite and (**b**) as a constant value. Improved performance of the advanced model compared to the constant filtration efficiency based on R^2^ and root mean square errors (RMSE) values.

A comparison in terms of coefficient of determination (R^2^) and root mean square errors (RMSE) over the NEDC, reveals the advantage of the DPF model compared to the constant filtration efficiency (Figure 14). The associated error yields as the average value from the comparison of estimated and measured by MSS soot emissions in every time step (Table 5).

### 3.3. Error Associated with the Sensor Model

The statistical model presented earlier (Methodology 2.4.3 Sensor Model) has three inputs: soot concentration, exhaust velocity and temperature. As expected from the operating principle of the resistive sensors, soot concentration is the governing parameter for sensor response, and the effect of temperature and velocity variations on the absolute response time is considerable only at low soot emissions. This behaviour is obvious from the results presented in Figure 9. The response of the sensor is calculated in the range of 1–14 mg/m^3^ (soot concentration). For low soot concentrations (e.g., at 3 mg/m^3^), an increase of exhaust temperature (e.g., from 180 °C to 260 °C) can create a significant increase in the absolute response time. In the case of higher soot levels (e.g., at 12 mg/m^3^), the absolute change in the response time is lower.

Each input is associated with the inaccuracies shown in Table 6. Regarding soot concentration, the introduced error is based on the actual soot measurement, which is the targeted accuracy of the soot model.

*δ_s_*: The inaccuracy of MSS is specified in the operation manual of the device [42]. It is derived from the resolution accuracy of the measurement device (5 μg/m^3^) and the dilution ratio accuracy of the conditioning unit (±2 + (dilution ratio (DR) × 0.5)%). For the current analysis, the dilution ratio was 10, with ±7% accuracy.

*δ_c_*: Temperature is measured via a Type K thermocouple, and the corresponding accuracy originates from the probe (0.75% × T) and the recording device (±0.25 °C).

*δ_v_*: The exhaust velocity is calculated based on the intake mass flow measured by a hot wire with ±2% accuracy, the fuel injection mass calculated from the ECU (its accuracy is considered same to the one of mass flow) and the pressure measured by a differential pressure sensor with *δ_p_* = 300 [Pa].

The propagated accuracy based on the above three variables is calculated at ±7% based on Equation (3), and this can be used as the inaccuracy introduced when a statistical model is used for the OBD model. The direct comparison of this result to other studies is not possible due to the absence of available quantitative results for the performance of every sub-model.

### 3.4. Error Associated with the Operation of the Resistive Soot Sensor

**Figure 15:**
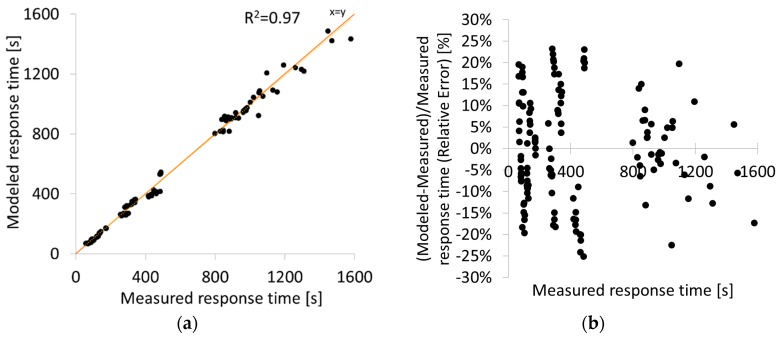
Measured compared to modeled response times in steady-state points, depicted as (**a**) x = y diagram and (**b**) relative error. The maximum error is ±25%.

Soot sensors have limited accuracy compared to laboratory measurement equipment. As an example, Khalek evaluated six different soot sensors in several steady-state points and concluded in ±60% accuracy in soot concentration measurement compared to a reference device [29]. This deviation is considered encouraging taking into account the small size and the low maturity level for the tested sensors [15]. In the current study, contrary to the previous example where the soot concentration was the sensor output, the response time is the output of the sensor. Thus, its accuracy should be calculated and discussed from this point of view based on several steady-state points. To this aim, 18 steady-state points were measured (Table 7). To compare the measured response time to a reference response time, the empirical function developed in Section 2.4.3 (Methodology, Sensor Model) was used to calculate the response time based on soot measurement of the reference instrument (MSS). The other two necessary input variables of the function (velocity and temperature) were calculated and measured, respectively, as described in Section 3.3. The measured and the calculated response times are compared in Figure 15a. The relative errors are presented in Figure 15b. The maximum relative error is ±25% and includes both the inaccuracy of the sensor and also the error introduced by the empirical function used for the modeled response time, which is ±7% according to Table 6. Therefore, the remaining error can be attributed to all parameters (except for the three inputs of the soot model) that affect the sensor operation. This can be expressed as the inaccuracy of the sensor, which is calculated at ±18%.

In an effort to explain this error, the analysis from Fragkiadoulakis et al. can be used [28]; they developed an advanced physical model that evaluates all mechanisms and variables governing the operation principle of resistive soot sensors. Despite that, based on this analysis, it is not possible to calculate the errors associated with the operation of the sensor and the accuracy of the sensor, but the governing mechanisms that result in this sensor inaccuracy can be clarified, and thus the sensor’s inaccuracy can be explained. The operating principle of the resistive soot sensors, as described in the methodology relies on the accumulation of particles on the sensor element and the resulting decrease of resistance between the two electrodes. The main deposition mechanisms are thermophoresis, electrophoresis, convective diffusion, inertial impaction and turbulence impaction. The primary inputs for all mechanisms are the exhaust soot concentration, velocity and temperature. However, except for these inputs, which are already included in the sensor model analysis, there are additional inputs which were proven to be significant for the physical model and therefore, significantly affect the performance of the sensor. Indicative examples are the particle size distribution, the sensor plate temperature, the electric field and the particle charge distribution. Therefore, it is important to bear in mind the possible bias in sensor response time originating by the complex sensor operation principle which results in its inaccuracy in the OBD model.

### 3.5. Overall OBD Model

The final implementation of the OBD model in the ECU includes all sub-models. Therefore, the calculated accuracy at this step reflects the accuracy of the DPF diagnosis. The propagation of the estimated error in all steps leads to a final inaccuracy of 28% (Table 8).

The above was calculated based on the examined scenario for an NEDC. WLTC and Real Driving Emissions (RDE) cycle, which represent more aggressive and closer to the real-world driving conditions, are expected to increase the individual inaccuracies and consequently, the overall error of diagnosis. On the other hand, the next step should be the understanding and the quantifications of possible improvements.

**Figure 16:**
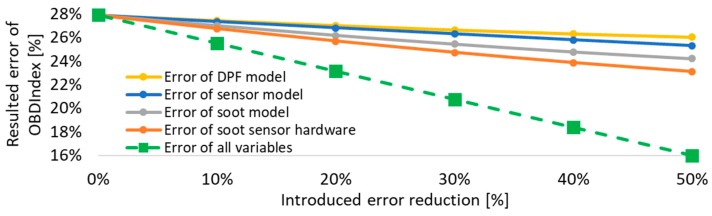
OBD index sensitivity analysis. A reduction of the inaccuracies of the sensor hardware and the soot model are the dominant factors for a reduced overall error.

A sensitivity analysis was performed as the next step for each of the above errors that contribute to the final OBD index error. Errors were reduced by up to 50% of the calculated values above. An improvement in sensor accuracy and a more accurate soot model will lead to a significant increase in the OBD accuracy of diagnosis. This agrees with the conclusion of a previous publication for a resistive soot sensor, which suggests that the highest effort should be put on the soot model calibration [36]. The accuracy of the sensor and DPF sub-models have a lower impact on the result compared to the previous factors (Figure 16) but can also be crucial for future tighter limits.

## 4. Conclusions

This study presents the challenges associated with an OBD model for diagnosis of a DPF using a resistive soot sensor. The described model consists of three sub-models, soot, DPF and sensor sub-models and the response time of a soot sensor. The model was evaluated for its accuracy in terms of percentage error on the estimated final OBD index over a transient operating cycle (NEDC). The error associated with the soot model for the estimation of the engine-out soot emissions was calculated to be ±25% (simulation without transient correction against measurement results). The accuracy of the DPF model was ±9% for the non-optimal scenario of constant filtration efficiency. The corresponding error for the advanced filtration efficiency developed in Exothermia/Axisuite was significantly lower (±5%). The sensor model, which implements the empirical function and estimates the sensor response time, leads to ±7% error associated with measurement inaccuracies on the sensor model. The sensor inaccuracy was estimated to be ±18% and is attributed to the mechanisms of soot deposition on the sensor element since they change even over the steady-state operation and thus affect the behavior of the sensor. The overall OBD index error based on a propagation analysis of the above errors was estimated to be ±28%.

The calculated error is expected to be higher over more transient operation during on-road operation. Nevertheless, improvements in every sub-model and sensor accuracy in its final production version will compensate for this effect. The performed sensitivity analysis indicates that the sensor accuracy improvement, along with a more accurate soot model, will significantly improve the performance of the OBD model. Finally, a fast sensor will allow for ECU debouncing strategies [43] and rejection criteria for adverse transient periods and thus successful diagnosis of a failed DPF under real driving conditions.

The reader should finally bear in mind that the error propagation was based on the results from a single driving cycle. Results of real-world driving conditions will be presented in a future study. Nevertheless, this analysis can be used as the basis for the improvement of the DPF diagnosis both for the needs of current legislation but also for the use of the resistive technology for future, more stringent limits or different legislation schemes such as the OBM [14].

## Figures and Tables

**Table 1:** European Union (EU) legislation limits and implementation dates for the first registration of vehicles.

	September 2015	September 2018
**Emission limits**	Euro 6a, b, NEDC	Euro 6c, d, WLTC
Particulate mass	4.5 mg/km	4.5 mg/km
Particle number	6 × 10^11^ #km ^a^	6 × 10^11^ #/km
**OBD limits**	OBD Euro 6–1	OBD Euro 6–2
Particulate mass	25 mg/km	12 mg/km ^b^
Particle number	No limit	No limit
Filter removal	DPF only	DPF only

^a^: 6 × 10^12^ #/km for GDI; ^b^: for all compression ignition engines and GDI; NEDC: New European Driving Cycle; WLTC: Worldwide harmonized Light-duty Test Cycles; OBD: on-board diagnostics; DPF: diesel particulate filter.

**Table 2:** Engine characteristics.

Feature	Value
Engine	Toyota 1.4 L
Fuel	Diesel
Displacement [cc]	1364
Fuel injection system	Common Rail, Direct Injection (DI)
Max Power [kW/rpm]	66/3800
Max Torque [Nm/rpm]	205/1400–2800
Cylinders	4
Gearbox	None
Emission standards	Euro 6b

**Table 3:** Operating point levels for the development of the soot model.

Engine Speed [rpm]	Engine Torque [Nm]
800	0
1200	10
1600	20
2000	60
2400	100
2800	140
3200	180
3600	

**Table 4:** Engine-out model variables and associated errors.

Variables	Source of Inaccuracy	Associated Error
Base soot model	Emission maps on transient operation	*δ_sv_* = 25%
Soot model with transient correction		*δ_sv_* = 2%

**Table 5:** DPF model’s variables and associated errors.

Variables	Source of Inaccuracy	Associated Error
DPF model: constant Filtration efficiency	Constant filtration efficiency	*δ_cf_* = 9%
DPF model: Axisuite filtration efficiency	Axisuite model	*δ_af_* = 5%

**Table 6:** Sensor model variables and associated errors.

Variables	Source of Inaccuracy	Associated Error
Exhaust soot concentration [mg/m^3^]	AVL 483 Micro Soot Sensor	*δ_s_* = 5 μg/m^3^
Exhaust temperature [°C]	Thermocouple Type K	*δ_c_* = 3.4 °C
Exhaust gas velocity [m/s]	Engine intake hot wire and ECU calculated value	*δ_v_* = 0.23%
Intake mass flow [kg/h]	Engine intake hot wire	*δ_m_* = 2%
Fuel injection mass [kg/h]	ECU calculated value	*δ_j_* = 2%
Exhaust Pressure [Pa]	Pressure sensor	*δ_p_* = 300 Pa
Sensor model	Empirical function	*δ_sm_* = 7%

**Table 7:** Steady-state levels for the tested sensors (Sensors 1–3).

Soot [mg/m^3^]	Velocity [m/s]	Temperature [°C]
1.5	12	180
4	24	240
14	38	

**Table 8:** OBD model variables and associated errors.

Variables	Source of Inaccuracy	Associated Error
Soot model	Transient operation	*δ_em_* = 25%
DPF model	Constant f.e.	*δ_dm_* = 9%
Sensor model	Inputs of empirical function	*δ_sm_* = 7%
Soot sensor accuracy	Sensor operation	*δ_s_* = 18%
Overall OBD model	Sub-models and sensor	*δ_OBD_* = 28%
